# Divergent responses of plant functional traits and biomass allocation to slope aspects in four perennial herbs of the alpine meadow ecosystem

**DOI:** 10.3389/fpls.2023.1092821

**Published:** 2023-03-01

**Authors:** Tianyang Zhou, Wentao Du, Jinniu Wang, Lin Zhang, Jing Gao, Ning Shi, Lihua Wang, Yan Wu, Binghui Tian

**Affiliations:** ^1^ Chengdu Institute of Biology, Chinese Academy of Sciences, Chengdu, China; ^2^ Northwest Institute of Eco-Environment and Resources, Chinese Academy of Sciences, Lanzhou, China; ^3^ Mangkang Biodiversity and Ecological Station, Tibet Ecological Safety Monitor Network, Changdu, China; ^4^ Institute of Tibetan Plateau Research, Chinese Academy of Sciences, Beijing, China; ^5^ Yangtze Eco-Environment Engineering Research Center, Shanghai, China; ^6^ College of Resources and Environment, Aba Teachers University, Wenchuan, China; ^7^ College of Resources and Environmental Sciences, Gansu Agricultural University, Lanzhou, China

**Keywords:** specific leaf area, isometric allocation, slope aspect, biotic and abiotic factors, alpine ecosystem

## Abstract

Slope aspect can cause environmental heterogeneity over relatively short distances, which in turn affects plant distribution, community structure, and ecosystem function. However, the response and adaptation strategies of plants to slope aspects *via* regulating their physiological and morphological properties still remain poorly understood, especially in alpine ecosystems. Here, we selected four common species, including *Bistorta macrophylla*, *Bistorta vivipara*, *Cremanthodium discoideum*, and *Deschampsia littoralis*, to test how biomass allocation and functional traits of height, individual leaf area, individual leaf mass, and specific leaf area (SLA) respond to variation in slope aspect in the Minshan Mountain, eastern Tibetan Plateau. We found that the slope aspect affected SLA and stem, flower mass fraction with higher values at southwest slope aspect, which is potentially related to light environment. The low-temperature environment caused by the slope aspect facilitates the accumulation of root biomass especially at the northeast slope aspect. *Cremanthodium discoideum* and *D. littoralis* invested more in belowground biomass in southeast and southwest slope aspects, although a large number of significant isometric allocations were found in *B. macrophylla* and *B. vivipara*. Finally, we found that both biotic and abiotic factors are responsible for the variation in total biomass with contrasting effects across different species. These results suggest that slope aspect, as an important topographic variable, strongly influences plant survival, growth, and propagation. Therefore, habitat heterogeneity stemming from topographic factors (slope aspect) can prevent biotic homogenization and thus contribute to the improvement of diverse ecosystem functioning.

## Introduction

Topography, as an important abiotic factor, strongly regulates ecosystem composition and structure across a relatively small distance, thereby influencing not just the ecological processes such as community assembly and soil nutrients cycling but also ecosystem function and services (i.e., productivity and carbon sequestration) from local to regional scale (Van de Water et al., 2002; Bennie et al., 2008; Burnett et al., 2008; Pereira et al., 2016; Pierick et al., 2021; Li et al., 2022). Slope aspect directly changes the distribution pattern of solar radiation, and therefore, soil temperature and moisture are altered accordingly (Burnett et al., 2008). For example, the most nutrient-rich soil and higher community diversity were found on the northern and southern slopes, respectively, in Eastern Qinghai–Tibetan plateau (Zhang et al., 2022). In addition, Singh (2018) demonstrated that the influence of slope aspect on the ecological process is highest in the mid-latitude region (i.e., 30° N–50° N) in the Northern Hemisphere. Currently, a limited number of studies have shown that plants in alpine ecosystem respond to fluctuations in micro-climate due to the variation in slope aspects. For instance, Li et al. (2021b) reported that higher leaf mass per area and small-leaved species were favored in the south slope aspect, while the reverse was found in the north slope aspect in Tibetan Plateau. In addition, slope aspect divergence in alpine ecosystems can also affect the pattern of alpine vegetation owing to the contrasting pattern of snow cover of accumulation or melting among slope aspects (Heegaard, 2002; Wu & Onipchenko, 2007). Hence, a better understanding of the slope aspect is critical for recognizing the ecological processes and functioning in alpine ecosystems.

To improve survival fitness and competition capacity, plants adjust their morphological and physiological traits in response to environmental fluctuation and biological interactions (Grassi and Bagnaresi, 2001; Wang et al., 2021). The variation in plant functional traits, which is a direct reflection of the adaptation of plants to environmental fluctuation, is controlled by phenotypical plasticity or genetic adaptation (Jung et al., 2010; Li et al., 2021b). Populations with higher trait variation probably have stronger adaption to rapid changes in environment and thus show higher stability (Albert et al., 2011). Adaptation of plant functional traits to environmental fluctuation follows the framework of the “leaf economic spectrum,” which summarizes a single major axis from the “quick-return” strategy in adequate resource availability to a “slow-return” strategy in resource-limited environments (Wright et al., 2004). For instance, specific leaf area (SLA) confers higher light capture capacity and use efficiency for plants, and plants with lower SLA are better able to adapt to adverse environmental conditions, such as a deficit of water and soil nutrients (Perez-Harguindeguy et al., 2016). Li et al. (2021b) reported that to better adapt to low-temperature environments, plants tend to have lower SLA to prevent water transpiration loss. Similarly, to enhance survival fitness under weak light environment, plants increase the SLA (Wang et al., 2021), as specific leaf area characterizes the light capture capability of plants (Wright et al., 2004; Poorter et al., 2012). However, leaf mass was associated with a conservative strategy, with higher values strongly associated with thicker cell walls and cuticles (Onoda et al., 2004; Li et al., 2021b). Plant height is related to competition for light, reproduction, and water transport, i.e., the more height investment, the greater plant support structure and light capture efficiency (Falster and Westoby, 2003). In addition, plant functional traits already emerged as an effective tool to predict ecosystem function (Lavorel and Garnier, 2002; Cadotte, 2017). However, how plant functional traits respond to the variation in micro-climate in a relative short distance caused by slope aspect is still inadequate.

Beyond functional traits, plants adapt to environmental variation by mediating biomass allocation. As an important ecological topic, biomass allocation strategies among different plant organs catch the attention of ecologists (Enquist and Niklas, 2002). The biomass allocation of plant represents the changes in growth and metabolism of plants (Hecht et al., 2019; Li et al., 2021a). For example, plants invest more aboveground biomass at the early ontogenetic stage while greater belowground biomass at the latter (Shipley & Meziane, 2002). Optimal partitioning and allometric partitioning these two theories are proposed to explain biomass allocation. Optimal partitioning theory states that plants invest more biomass in the organs that acquire the most limiting resources to enhance their performance (McCarthy and Enquist, 2007; Chave et al., 2014). For instance, when light or CO_2_ is restricted, plants allocate relatively more biomass to aboveground organs but more biomass to roots in case of deficient water or soil nutrient (Poorter et al., 2012). Allometric theory suggests that growth rates of organisms are stable across different sizes, and biomass accumulation in different organs has an anisotropic relationship, determined by a power function (Enquist and Niklas, 2002; Coomes, 2006). Although the allometric scaling patterns have been well documented, most studies have found that isometric biomass allocation was common in the natural ecosystems (Dolezal et al., 2021). For example, a study conducted on the Tibetan Plateau reported an isometric relationship between aboveground biomass (AGB) and belowground biomass (BGB) at the community level across various grassland ecosystem types (Yang et al., 2010). For that, both optimal partitioning theory and allometric theory can explain plant biomass allocation simultaneously to a certain extent (Gedroc et al., 1996; Ma and Wang, 2021). The biotic and abiotic factors can also affect the accumulation of biomass in addition to the biomass allocation strategies (Poorter et al., 2012; Reich et al., 2014). Leaf economic spectrum also described that plants with higher SLA facilitate the aboveground biomass accumulation. Therefore, plant total biomass is driven by biotic and abiotic factors. Yet, how biotic and abiotic factors, especially for slope aspect, explain the variation in total biomass and biomass allocation of alpine plants and their relative importance is still unclear.

The Tibetan Plateau has experienced a higher degree climate warming effect than the global average. It is the world’s highest plateau, with 64% of the region occupied by alpine grasslands that provide essential ecosystem services for resident livelihood and community development in the area (Ma et al., 2017). In particular, the eastern part of the Tibetan Plateau is characterized by thermally restricted and low material turnover rate, where plants grow in harsh habitat with limited resources compared to other regions (Körner, 2003; Wang et al., 2016). To effectively maintain the ecosystem functions of grassland in this region, a key step is to comprehensively understand how micro-climate variability influences plant life strategy (e.g., functional traits and biomass allocation). In the present study, we collected four perennials in alpine meadow ecosystems as target species from three slope aspects at two mountains, i.e., *Bistorta macrophylla*, *Bistorta vivipara*, *Cremanthodium discoideum*, and *Deschampsia littoralis*. We aimed to address these main questions: 1) what strategies are plant functional traits adopting to coordinate micro-environmental variation caused by slope aspect; 2) how does the mass fraction of plant organs (i.e., root, stem, leaf, and flower) have specific response to slope aspects; and 3) between biotic and abiotic factors, whose impact is more pronounced in regulating total biomass, and whether these effects are consistent across species.

## Materials and methods

### Study area

**Figure 1:**
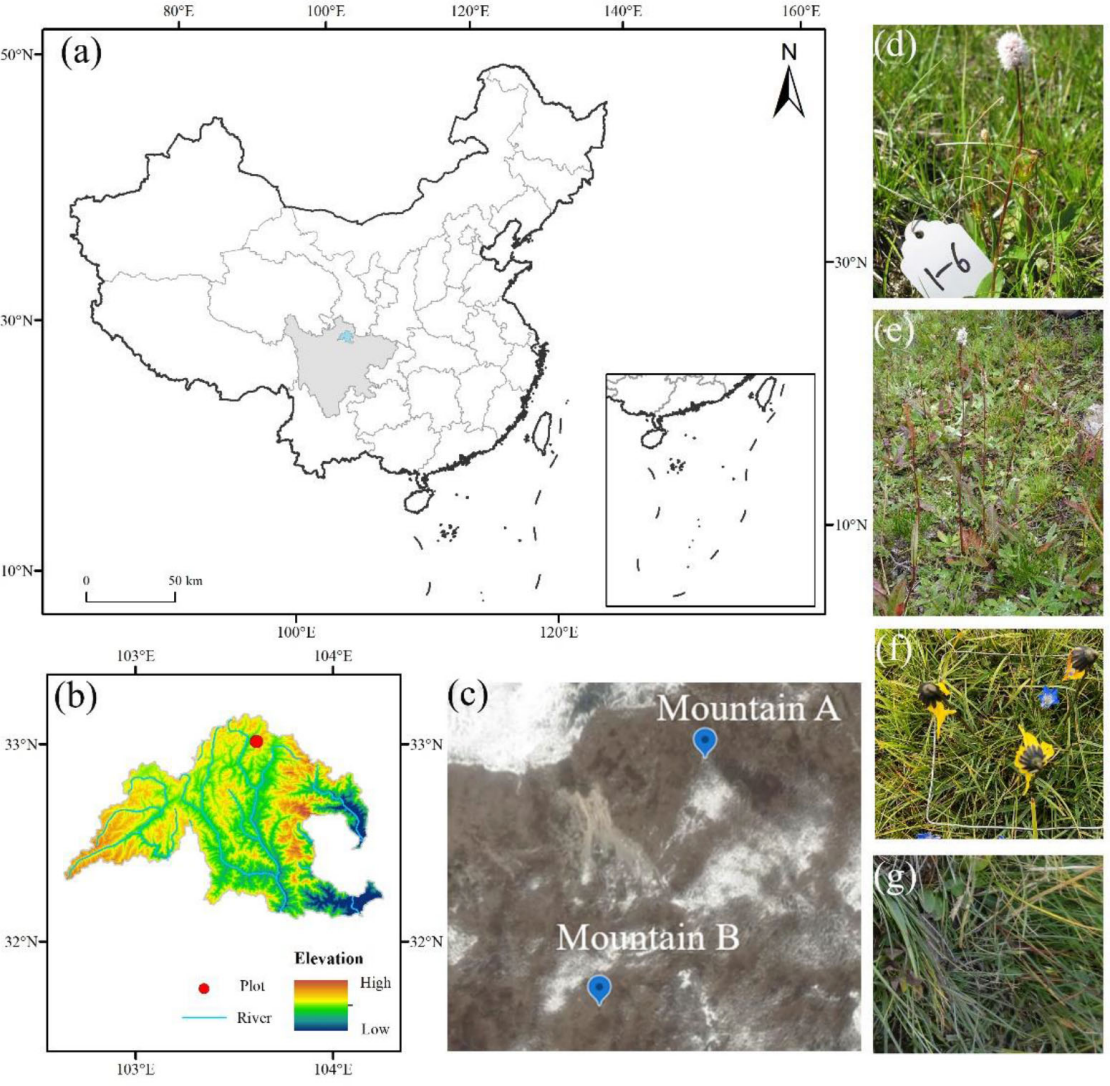
Study site **(C)** at mountain Kaka and its location at Songpan county **(B)** and China **(A)**. Four target species are selected in this study, including *Bistorta macrophylla* **(D)**, *Bistorta vivipara* **(E)**, *Cremanthodium discoideum* **(F)**, and *Deschampsia littoralis* **(G)**.

This study was conducted in Mountain Kaka (103° 40′ N, 32° 59′ E, **Figure 1**), located in the middle section of the Minshan Mountains, eastern Tibetan Plateau. The area forms the headstream of Minjiang and Fujiang Rivers, sitting in west Sichuan Province, China (Wang et al., 2021). The vegetation of this region has distinct altitudinal zonation and horizontal distribution with strong floristic transition and abundant plant species. Meanwhile, this region is characterized by contrasting distribution of solar radiation due to the complex undulating terrain (Shi et al., 2022). The mean annual, January (coldest month), and July (warmest month) air temperature is 5.7°C, −7.9°C, and 9.7°C, respectively, and the annual precipitation is 720 mm with peaks during June to August. The mean annual sunshine period is approximately 1,827 h. The soil is classified as Mat-Gryic Cambisol, with the pH value ranging from 6.21 to 7.11 in surface soil (0–10 cm). The grassland type is a typical alpine meadow dominated by *Ranunculus tanguticuz*, *Pedicularis kansuensis*, *Pyrethrum tatsienense*, *B. macrophylla*, *B. vivipara*, and *C. discoideum* (Wang et al., 2021).

### Experimental design and sampling

During the growing season (mid-June) in 2016, field investigation was conducted at two sites, namely, Mountain A (103° 40′ 16″E, 32° 58′ 57″N, 3828 m a.s.l.) and Mountain B (103° 40′ 12″E, 32° 58′ 48″N, 3797 m a.s.l.). Two surrounding sample plots were set up horizontally at 30 m vertically downward from the summit of two mountains, respectively, dividing into three slope aspects according to the ridge trend, i.e., northeast (30°E), southeast (45°W), and southwest (45°E) slope aspect (**Figure 1**). Since a large number of dwarf shrubs were distributed at the northwest slope aspect, no sampling plots were set up at this slope aspect to prevent the influence of shrubs on the growth of target species. Three subplots (5 m × 5 m) with 10-m spacing between each other were set up in an area with a relatively even distribution of vegetation in each slope aspect. Four common species, including *B. macrophylla*, *B. vivipara*, *C. discoideum*, and *D. littoralis*, as target species were collected, since they were distributed mainly in southwest China with a range of altitude from 1,200 to 5,400 m with different life forms, including C3 (*B. macrophylla* and *B. vivipara*) and C4 species (*C. discoideum* and *D. littoralis*). However, not all species were present at all slope aspects, and we did not find *C. discoideum* and *D. littoralis* at the northeast slope aspect at mountain A. For each species, 20–30 fully mature individuals including intact roots were sampled. All the fresh samples were stored in iceboxes and transferred to the laboratory for further measurements.

### Leaf traits, biomass, and soil properties measurement

In total, we sampled 501 plant individuals from two mountains (**Supplementary Table S1**). For each individual, different organs of root, stem, leaf, and flower were separated, and plant height (cm), individual leaf area (ILA, cm^2^), individual leaf mass (ILM, g), and specific leaf area (SLA, cm^2^ g^−1^) were measured following the standard protocols (Perez-Harguindeguy et al., 2016). Sampled fresh leaves were scanned by Canon Scan Gear and calculated by Photoshop CS4 and Matlab 7.9 for their leaf area (Wang et al., 2021). ILA was defined as the ratio of the total leaf area to the number of leaves of each plant individual. Plant height was measured by a ruler. Then, all the samples were dried in an oven at 65°C for a minimum of 48 h. Root mass fraction, stem mass fraction, leaf mass fraction, and flower mass fraction were calculated as the ratio of their respective dry masses to the total dry biomass. ILM was defined as the total leaf dry mass to the number of leaves. SLA was calculated as the ratio of leaf area to leaf dry weight. Total biomass is the sum of belowground biomass (i.e., root mass) and aboveground biomass (i.e., stem mass, leaf mass, and flower mass).

To evaluate the effect of soil properties on functional traits and biomass, we collected soil samples by using a soil auger and an aluminum specimen box. For each subplot, three soil samples were collected from the surface to a depth of 10 cm and mixed into one composite sample. In total, 27 soil samples were collected for each mountain. Soil properties, including soil temperature at the surface (ST, °C) and at 5 cm below the surface (ST5, °C), bulk density (BD, g cm^−3^), soil water content (SWC, %), nitrate nitrogen content (NNC, mg kg^−1^), ammonium nitrogen content (ANC, mg kg^−1^), soil organic carbon (SOC, g kg^−1^), total nitrogen (TN, g kg^−1^), total phosphorus (TP, g kg^−1^), and available phosphorus (AP, mg kg^−1^), were measured (**Table 1**). Soil temperature was measured by a button thermometer (iButton-TMEX RTE). Soil bulk density was measured by using the cutting ring technique, while the oven-drying method was used for soil water content. The indophenol blue colorimetric and ultraviolet spectrophotometry methods were used to measure ammonium nitrogen content and nitrate nitrogen content, respectively (Wang et al., 2021). Soil organic carbon and total nitrogen were measured by using the dry combustion method on an elementary analyzer. Total phosphorus and available phosphorus were measured by extraction with 0.5 M sodium hydroxide sodium carbonate solution (Wang et al., 2016).

**Table 1:** The basic information about soil properties at two mountains.

Mountain	A			B		
Slope aspect	Northeast	Southeast	Southwest	Northeast	Southeast	Southwest
ST5	11.03 ± 0.41a	10.03 ± 0.15b	11.28 ± 0.17a	12.91 ± 0.27b	14.13 ± 0.36a	14.77 ± 0.30a
ST	13.10 ± 0.43a	9.17 ± 0.39c	10.79 ± 0.30b	8.90 ± 0.18c	11.66 ± 0.32a	10.24 ± 0.32b
BD	0.69 ± 0.02c	0.74 ± 0.03b	0.91 ± 0.01a	0.92 ± 0.03a	0.93 ± 0.05a	0.91 ± 0.03a
SWC	39.43 ± 1.57a	44.92 ± 8.69a	38.38 ± 3.28a	39.55 ± 1.19ab	41.96 ± 1.28a	33.79 ± 4.73b
NNC	13.78 ± 1.94a	8.32 ± 4.58a	17.19 ± 6.03a	3.00 ± 1.29b	10.20 ± 6.05a	16.82 ± 1.92a
ANC	3.96 ± 0.90b	12.67 ± 5.16a	7.02 ± 1.94a	5.56 ± 0.70a	5.12 ± 0.94a	9.08 ± 3.06a
SOC	32.81 ± 1.28a	36.82 ± 7.49a	30.79 ± 3.98a	30.48 ± 1.34b	35.51 ± 2.29a	33.51 ± 2.38ab
TN	2.82 ± 0.17a	3.15 ± 0.55a	2.89 ± 0.42a	2.78 ± 0.18b	3.14 ± 0.12a	3.07 ± 0.23ab
TP	0.68 ± 0.03a	0.75 ± 0.11a	0.75 ± 0.10a	0.73 ± 0.01b	0.78 ± 0.04b	0.95 ± 0.01a
AP	8.53 ± 0.45a	9.46 ± 0.80a	10.88 ± 2.28a	7.79 ± 0.61b	8.95 ± 0.35b	12.47 ± 0.77a

ST and ST5, soil temperature at the surface and at 5 cm below the surface; BD, bulk density; SWC, soil water content; NNC, nitrate nitrogen content; ANC, ammonium nitrogen content; SOC, soil organic carbon; TN, total nitrogen; TP, total phosphorus; AP, available phosphorus. Different letters indicate significant differences for soil properties in different slope aspects of each mountain.

### Statistical analysis

First, the difference in soil properties among slope aspect for each mountain was tested by one-way ANOVA. Second, the coefficient of variation was used to quantify the variation in functional traits for each species, which is defined as the ratio of standard deviation to the mean of each functional trait. Third, one-way ANOVA was used to assess the effect of the slope aspect on the variations in functional traits and biomass allocation for each species. All response variables were log-transformed to best meet model assumptions. Fourth, we used standardized major axis analysis (SMA) to explore the biomass allocation between above- and below ground, and the effect of the slope aspect on variation in biomass allocation strategy. The allometric equation of the form Y = c·X^a^ was used to test biomass allocation strategy among slope aspects, where Y is the aboveground biomass (i.e., sum biomass of stem, leaf, and flower), X is the belowground biomass, and a and c are allometric coefficients. The equation was logarithmically transformed into a linear equivalent, ln(Y) = ln(c) + a·ln(X) (Niklas & Enquist, 2001).

Finally, we used the structural equation model (SEM) to test the direct and indirect effect of both biotic (i.e., height, ILA, ILM, and SLA) and abiotic factors (i.e., slope aspect, soil properties) on the total biomass of each species. To reduce the dimensionality of soil properties, we ran principal component analysis (PCA) for 10 soil properties. The first three PCA axes explained 84% of the total variation (**Supplementary Table S2**), which were used in the SEM. The first PC axis (PC1) described lower SWC and higher TP and AP. The second PC axis (PC2) described lower NNC, SOC, and TN, and the third PC axis (PC3) described lower ST. We constructed a hypothetical causal model, which includes several direct paths from biotic and abiotic factors to total biomass and indirect path from abiotic factors to total biomass *via* biotic factors (**Supplementary Figure S1**). The slope aspect was treated as a regular numeric variable and coded as 1 (northeast), 2 (southeast), and 3 (southwest) before formulating a structural equation model. To optimize SEM, we removed the non-significant paths (*p* > 0.05). Then, Fisher’s C statistic (*p* > 0.05) and Akaike’s information criterion (AICc) were used to estimate the fit of the global model (Shipley, 2009). All data analyses were performed in R v.3.6.0 (R Core Team, 2019). SMA, PCA, and SEM analysis were performed using the R package “smart” (Warton et al., 2012), “vegan” (Oksanen et al., 2013), and ‘‘piecewiseSEM’’ (Lefcheck, 2016), respectively.

## Results

### Intraspecific trait variation and the variation in functional traits at different slope aspects

**Figure 2:**
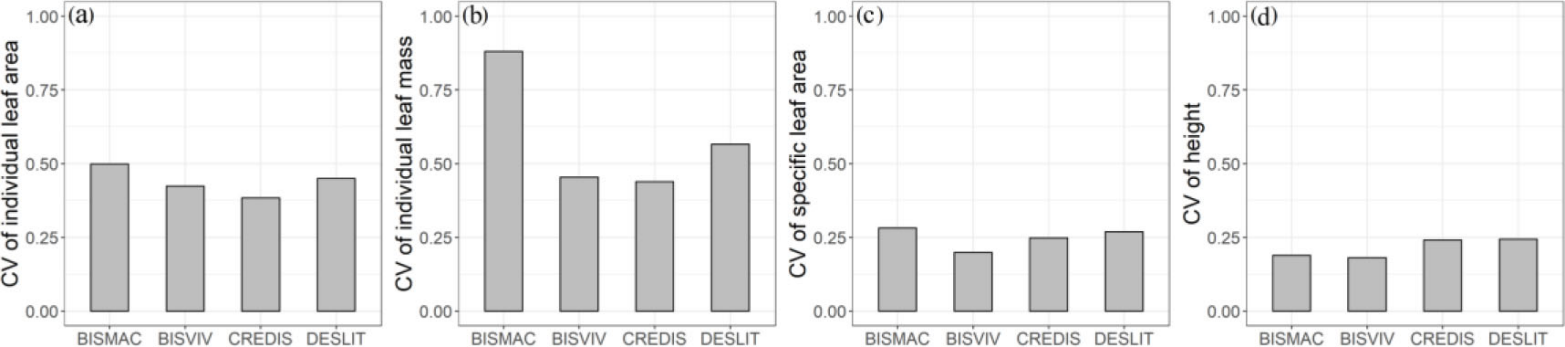
Intraspecific trait variation in individual leaf area **(A)**, individual leaf area **(B)**, specific leaf area **(C)**, and height **(D)** for BISMAC (*Bistorta macrophylla*), BISVIV (*Bistorta vivipara*), CREDIS (*Cremanthodium discoideum*), and DESLIT (*Deschampsia littoralis*).

Intraspecific trait variation in individual leaf mass had the largest magnitude, followed by individual leaf area, SLA, and height in turn. The trait CV values of four species in individual leaf mass ranged from 43.88% to 88.05% (**Figure 2**; **Supplementary Table S3**). The CV values of individual leaf area, SLA, and height were ranged from 38.40% to 49.89%,19.86% to 28.12%, and 18.05% to 24.36% (**Figure 2**; **Supplementary Table S3**), respectively.

**Figure 3:**
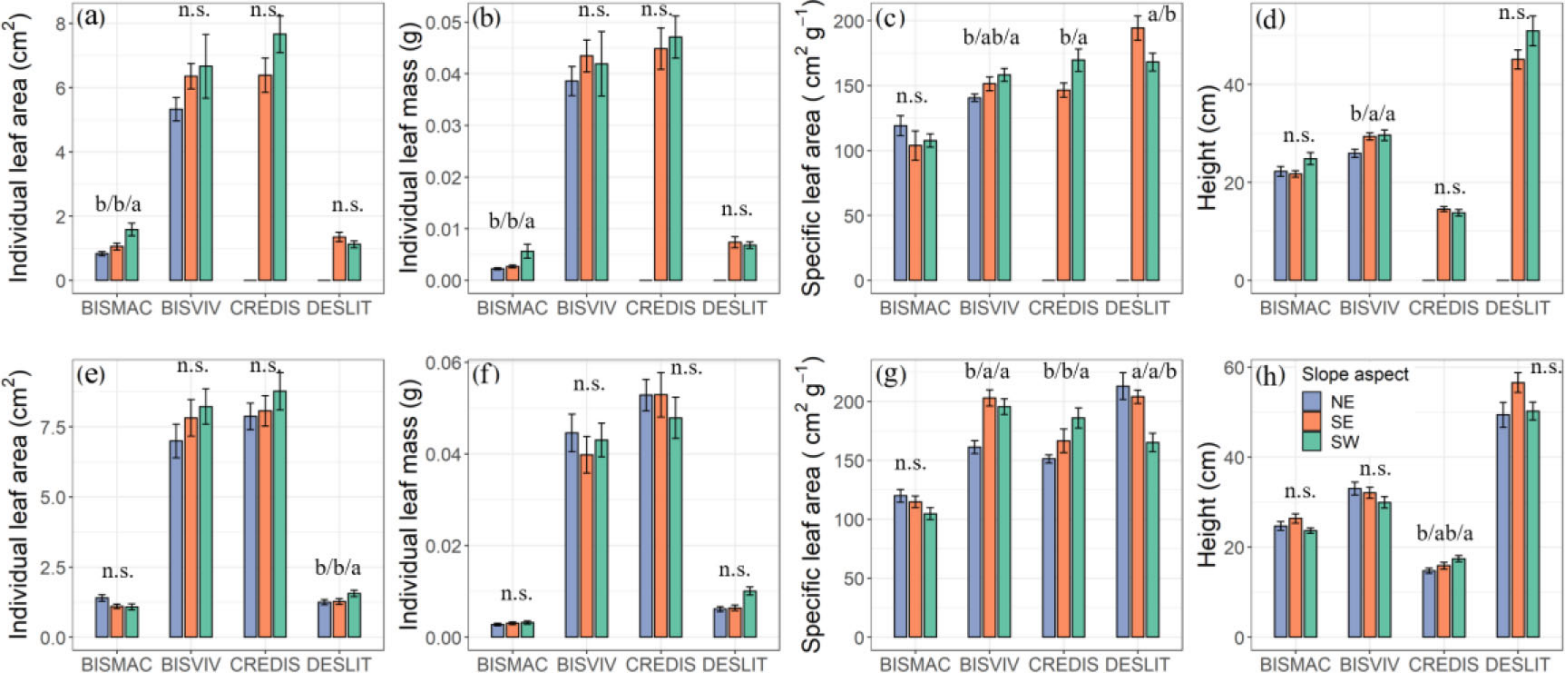
Effect of slope aspect on functional traits at mountain A **(A–D)** and mountain B **(E–H)**. NE, SE, and SW refer to northeast, southeast, and southwest slope aspect, respectively. Letters indicate significant differences (n.s., not significant). Values are mean ± SE. BISMAC, BISVIV, CREDIS, and DESLIT are the abbreviation of *Bistorta macrophylla*, *Bistorta vivipara*, *Cremanthodium discoideum*, and *Deschampsia littoralis*, respectively.

Slope aspects had significant effect on the functional traits of four species divergently but species specific (**Figure 3**). We found the highest SLA of BISVIV and CREDIS, and the lowest SLA of DESLIT existed on the southwest slope aspects in both mountains (**Figures 3C, G**). However, the slope aspect had a minor effect on individual leaf area and mass, and height. The lowest and highest individual leaf area were observed for BISMAC (0.83 ± 0.07 cm^2^, 1.58 ± 0.20 cm^2^) and DESLIT (1.24 ± 0.09 cm^2^, 1.56 ± 0.12 cm^2^) at northeast and southwest slope aspects, respectively (**Figures 3A, E**). In addition, the lowest (0.0022 ± 0.0002 g, **Figure 3B**) and highest (0.0056 ± 0.0013 g, **Figure 3B**) individual leaf mass of BISMAC were presented in northeast and southwest slope aspects, respectively. Similarly, the heights of BISVIV and CREDIS were found to increase in terms of slope aspects from the northeast, southeast, to the southwest (**Figures 3D, H**; **Supplementary Table S4**).

### The difference in mass fraction of each organ among slope aspects

A relatively higher root mass fraction of BISMAC (54.74%–67.07%) and BISVIV (60.90%–79.00%) but lower leaf mass fraction (4.65%–8.95%, 6.85%–12.26%) had been observed. CREDIS and DESLIT reflected lower root mass fraction (16.65%–25.30%, 12.62%–40.88%) and higher leaf mass fraction (38.81%–46.57%, 42.63%–58.42%). A higher flower mass fraction existed in CREDIS (20.82%–27.27%), followed by BISMAC (10.10%–15.16%), BISVIV (1.48%–4.15%), and DESLIT (2.40%–5.51%). The stem mass fraction of BISMAC, BISVIV, CREDIS, and DESLIT ranged from 18.03% to 22.52%, 10.16% to 24.49%, 10.75% to 14.55%, and 13.80% to 25.77%, respectively (**Supplementary Table S5**).

**Figure 4:**
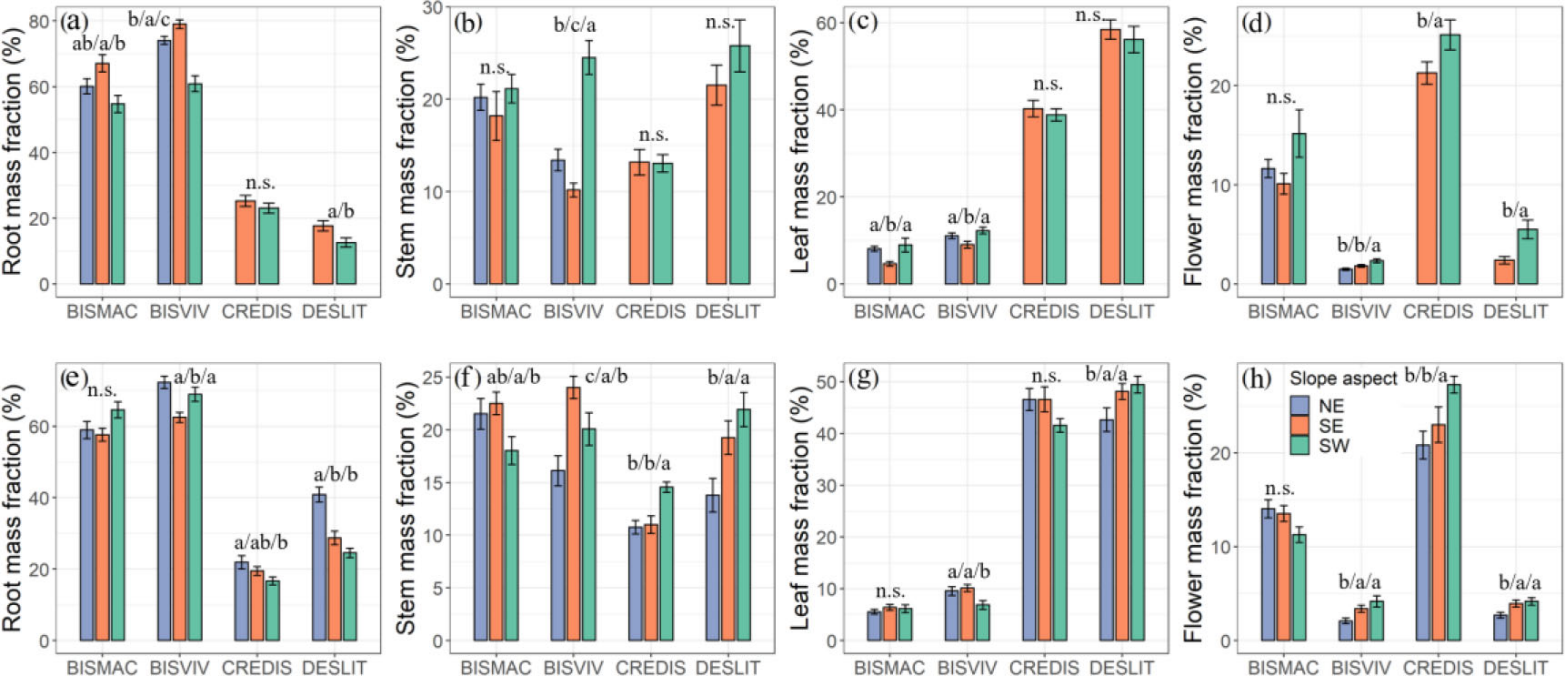
Effect of slope aspect on mass fraction at different organs at mountain A **(A–D)** and mountain B **(E–H)**. NE, SE, and SW refer to northeast, southeast, and southwest slope aspects, respectively. Letters indicate significant differences (n.s., not significant). Values are mean ± SE. BISMAC, BISVIV, CREDIS, and DESLIT are the abbreviation of *Bistorta macrophylla*, *Bistorta vivipara*, *Cremanthodium discoideum*, and *Deschampsia littoralis*, respectively.

One-way ANOVA indicated that the mass fraction of each organ of BISVIV and DESLIT was significantly affected by the slope aspect (*p* < 0.05, **Figure 4**). The slope aspect had a minor effect on both leaf mass fraction of CREDIS and flower mass fraction of BISMAC (**Figure 4**). The effect of slope aspect on mass fraction was not consistent between the two mountains. For example, the slope aspect exerted a significant effect on root and leaf mass fraction of BISMAC at mountain A but not at mountain B (*p* < 0.05, **Figure 4**). Similarly, slope aspect had a minor effect on root and stem mass fraction of CREDIS at mountain A but reflected a significant effect on them at mountain B (*p* < 0.05, **Figure 4**; **Supplementary Table S5**).

### The difference in above- and belowground biomass allocation among slope aspects

**Figure 5:**
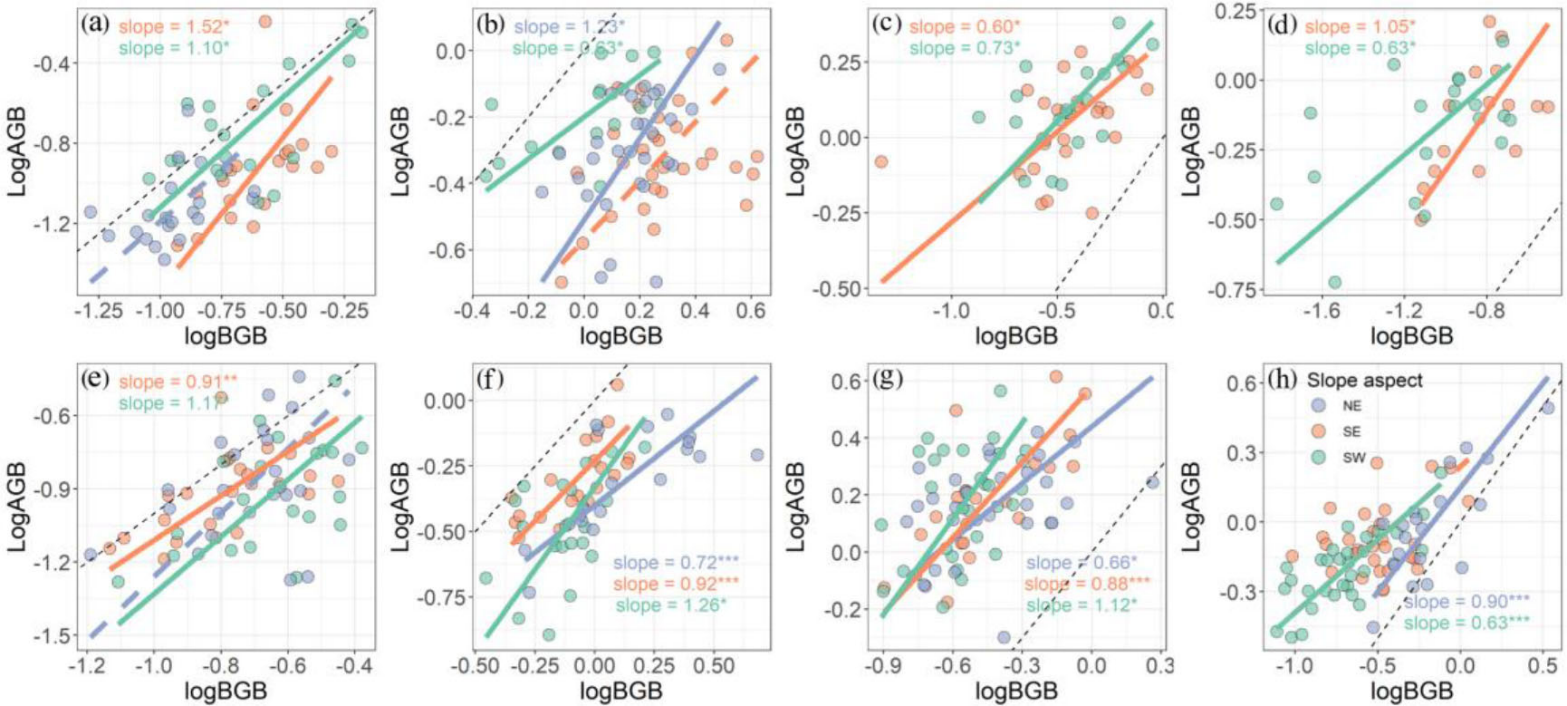
Effect of slope aspect on plant above- and belowground biomass allocation strategies at mountain A **(A–D)** and mountain B **(E–H)**. NE, SE, and SW refer to northeast, southeast, and southwest slope aspect, respectively. **(A, E)** *Bistorta macrophylla*; **(B, F)** *Bistorta vivipara*; **(C, G)** *Cremanthodium discoideum*; **(D, H)** *Deschampsia littoralis*. Slope refers to the regression slope of each model with asterisks indicating significance (*p < 0.05, **p < 0.01, and ***p < 0.001). Solid and dashed lines indicate the significant and non-significant relationships. The black dotted line is a line with slope equal to 1.

Standardized major axis analysis revealed that the above- and belowground biomass allocation of these four species at different slope aspects shared a common slope (*p* > 0.05, **Figure 5**; **Supplementary Table S6**). We found that most of the species presented an isometric growth relationship between AGB and BGB at different slope aspects (**Figure 5**). However, the slope of BGB against AGB for CREDIS was significantly <1 at the southeast of mountain A and northeast of mountain B (*p* < 0.01, **Supplementary Table S6**). Similar results for DESLIT were observed at southwest slope aspect of both mountains A and B (*p* < 0.05, **Supplementary Table S6**). In addition, we only found that the regression slope of SMA was significantly >1 for BISMAC at the southeast slope aspect at mountain A (*p* = 0.049, **Supplementary Table S6**).

### Biotic and abiotic factors jointly explain the variation in the total biomass of each species

**Figure 6:**
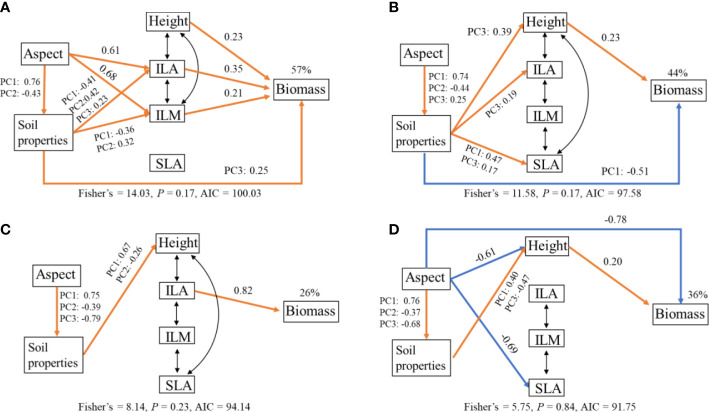
Structural equation model relating total biomass of *Bistorta macrophylla* **(A)**, *Bistorta vivipara* **(B)**, *Cremanthodium discoideum* **(C)**, and *Deschampsia littoralis* **(D)** to slope aspect (Aspect), soil properties, height (Height), individual leaf area (ILA), individual leaf mass (ILM), and specific leaf area (SLA) in alpine grassland. The coefficients are standardized prediction coefficients for each causal path. Orange and blue lines represent significant positive and negative associations, respectively, and black bi-directional arrows indicate correlations. PC1, PC2, and PC3 indicate the first three PCA axis of soil properties. The percentages above Biomass indicate the proportion of variance explained.

These four SEMs provided a good fit to the data and accounted for 57%, 44%, 26%, and 36% of the variation in total biomass for BISMAC, BISVIV, CREDIS, and DESLIT, respectively (**Figure 6**; **Supplementary Tables S7–S10**). The relative importance of biotic and abiotic factors in driving total biomass for four species was not consistent. For BISMAC, soil properties, height, ILA, and ILM had positive direct effects on total biomass. Slope aspect and soil properties affected biomass indirectly *via* ILA and ILM (**Figure 6A**). For BISVIV, soil properties and height presented a negative and positive effect on biomass, respectively. Soil properties also played a positive indirect effect on biomass *via* height, ILA, and ILM. Slope aspect exerted an indirect effect on biomass *via* soil properties (**Figure 6B**). For CREDIS, we found that only ILA exerted a positive direct effect on biomass. Abiotic factors had a minor effect on biomass (**Figure 6C**). For DESLIT, height played a significant positive role on biomass. Slope aspect had a negative direct effect and an indirect effect *via* height and soil properties on biomass (**Figure 6D**).

## Discussion

### Intraspecific trait variation and effects of slope aspect on variation in plant functional traits

Variation in intraspecific plant traits relates to the phenotypic trait plasticity of species, which is determined by environmental conditions and genetics simultaneously (de Bello et al., 2011; Violle et al., 2012; Dong et al., 2020; Li et al., 2021a). Our results showed that BISMAC had higher intraspecific trait variation, especially for ILA and ILM, which suggested that BISMAC may have a relatively higher survival fitness in a stressful alpine ecosystem. However, the intraspecific trait variation in SLA and height is smaller than that of ILA and ILM of each species. The similarity and lower variation in SLA and height may arise as a consequence of the habitat filter in harsh environmental conditions leading to a similar life strategy for different species (Violle et al., 2012; Dong et al., 2020). Our study indicated that the slope aspect had a significant effect on functional traits, especially for SLA (**Figure 3**). As an important functional trait in leaf economic spectrum, SLA is closely related to resource acquisition and use efficiency (Wright et al., 2004). The significant difference in SLA of BISVIV and CREDIS across slope aspects at both mountains may attribute to two potential reasons. First, limited light at southwest slope aspect allows plants to have a larger SLA to enhance light use efficiency, which is in line with a recent study (Li et al., 2021a). Meanwhile, to increase light use efficiency, plant also tend to possess larger leaf area. A larger leaf area of BISVIV and CREDIS was also found at the southwest slope aspect, despite being non-significant, which may support this scenario to a certain extent for light as a limiting factor at this slope aspect. In addition, we also found higher individuals of BISVIV and CREDIS at the southwest slope aspect at mountains A and B, respectively, which shows that plants allocate more towards photosynthesis production in height to strengthen their competitive ability for light resources (Dolezal et al., 2021). Second, the variation in temperature leads to a change in SLA. We found that a smaller SLA of BISVIV and CREDIS occurred at northeast slope aspect at mountain B. As the lower temperature forces high mountain plants to shift life strategy to become more conservative with lower photosynthetic rate and evapotranspiration rate, to enhance their survival fitness, it can explain why a smaller SLA emerged at the northeast slope aspect with the lowest temperature in this study. Interestingly, but not surprisingly, we found that the variation in SLA of DESLIT among slope aspects presented the opposite pattern to BISVIV and CREDIS, which highlighted the heterogeneity in species response to the same environment (Niinemets, 2006; Cheng and Niklas, 2007; Valladares and Niinemets, 2008). Functional traits can effectively affect growth of individuals and even predict ecosystem functioning like productivity and stability (Cadotte, 2017; He et al., 2019). In majority of the previous studies, community-weighted traits are usually used to predict ecosystem function (Tjoelker et al., 2005; Craven et al., 2018). However, our results provide a new insight that the predictive capability of functional traits for ecosystem functioning may be enhanced when we account for the variation in functional traits under microclimates.

### Effect of slope aspect on mass fraction and above- and belowground biomass allocation

Similarly, slope aspect played a significant effect on mass fraction among organs (**Figure 4**). To maximize survival and optimize growth in the alpine ecosystem, plants generally allocate more biomass to belowground part (Dolezal et al., 2021). We found that a higher root mass fraction occurred at the low-temperature slope aspect (**Figure 4**). The root mass fraction of CREDIS and DESLIT ranged from 16.65% to 25.30%, and 12.62% to 40.88%, respectively, which is consistent with a previous study reporting root mass fraction values of plants between 10% and 50% (Poorter et al., 2012; Poorter et al., 2015). However, in our study, the root mass fraction of BISMAC and BISVIV far exceeded this range, especially at the northeast slope aspect. In particular, compared to the south slope aspect, the north slope aspect in northern hemisphere receives less solar radiation, thus resulting in a lower-temperature condition in this slope aspect (Singh, 2018). As such, the slower depletion of carbohydrates and turnover of root stem from colder environments lead to higher accumulation of root mass (Davidson, 1969; Gill and Jackson, 2000; Yang et al., 2010; Pierick et al., 2021). The higher stem mass fraction mainly emerged at the southwest slope aspect, as mentioned above, plants invest more biomass to the stem to increase asymmetric light competition. Consistent with results on the effect of slope aspect on functional traits, the variation in stem mass fraction among slope aspects did not follow a similar pattern between four species, which again highlighted their species-specific response (Niinemets, 2006; Cheng and Niklas, 2007; Valladares and Niinemets, 2008). It also emphasizes the importance of biodiversity for ecosystems with hyper-diverse communities of species with different strategies for responding to environmental fluctuations that will reduce overall community fluctuations through asynchronous responses among populations and thus maintain ecosystem function (also known as species asynchrony, Loreau and de Mazancourt, 2008). Finally, flower mass fraction was also higher at the southwest slope aspect. Investing more resources into flowers in light-limited areas will help plants maintain a higher reproductive capacity in adverse environments (Leck et al., 2008).

Plants adjust their biomass allocation strategies to maintain necessary physiological activities, achieve normal growth, and improve environmental adaptability (Shipley and Meziane, 2002; Mensah et al., 2016). The allocation of plant biomass among organs is not only driven by environmental conditions but also genetics (Poorter et al., 2012; Pallas et al., 2016). We found that most species presented an isometric relationship between above- and belowground biomass at several slope aspects, which is in line with a research focus on community level (Yang et al., 2010). However, both CREDIS and DESLIT presented an allometric relationship, investing more biomass belowground than aboveground at several slope aspects (**Figure 5**). Meanwhile, our results are also consistent with a study that alpine plants are threatened by low temperatures, even though a shallow snow cover can alleviate the low temperature stress somehow (Dolezal et al., 2016). Additionally, we also found that BISMAC invested more biomass to aboveground at the south east slope aspect, while it invested equal biomass between above- and belowground at the south west slope aspect, which support the optimal partitioning theory that plants are able to achieve optimal acquisition of resources by regulating biomass allocation strategies (Gedroc et al., 1996). It is worth to note that we did not take the density effect into account in this study. For instance, plants tend to invest more biomass to aboveground for a higher competitive capacity for light resources (Poorter et al., 2012; Sun et al., 2021; Tang et al., 2021; Tao et al., 2021). Therefore, the density effect on biomass allocation needs to be considered carefully in future studies.

### Biotic and abiotic factors jointly drive dominant species population biomass

Our results indicated that both biotic and abiotic factors drove total biomass of plants simultaneously, while their relative importance varied between the four species (**Figure 6**). The plant height can explain the large variation in biomass, which is in line with recent studies from aquatic plant communities (Gustafsson and Norkko, 2019). The increase in plant height facilitates competition for light resources, which in turn increases productivity (Westoby, 1998; Diaz et al., 2004). For the same reason, the increase in individual leaf area enhanced the assimilation efficiency for biomass accumulation, which supports the leaf economic spectrum hypothesis that exploitative traits tend to have higher productivity (Wright et al., 2004). Unexpectedly, SLA had a neutral effect on biomass. Probably, some unmeasured traits, such as root traits, may increase the traits explanation of biomass in alpine ecosystems with lower temperature stress. In addition, interspecies relationships can also be critical for biotic regulation of biomass allocation, depending on their positive facilitation or negative competition. Thus, these issues should be taken into consideration in future studies to ascertain the relative importance and contribution of biotic and abiotic factors, respectively. In addition, our study also showed that abiotic factors have both direct and indirect effect on biomass. For example, slope aspect affected total biomass directly and indirectly *via* height for DESLIT. Moreover, consistent with recent research, we found that slope aspect significantly affected soil properties, which thus mediated plant biomass indirectly (Li et al., 2021b; Zhang et al., 2022). Similarly, we also found direct and indirect effects of soil properties on biomass *via* different path between four species. In summary, our results emphasize the importance of slope aspect for alpine plants not just about plant traits but also biomass allocation regulating their plasticity to merge biotic and abiotic ways.

## Conclusion

This study provides a comprehensive analysis of how functional traits, mass fraction, and biomass allocation of plants respond to slope aspect. Meanwhile, we revealed how biotic and abiotic factors drive the total biomass of four dominant species in the alpine meadow ecosystems. Our findings show that SLA, stem, and flower mass fraction were significantly affected by slope aspect, which might be potentially related to the light environment. Whereas higher root mass fraction is mainly linked to the low-temperature condition caused by the slope aspect, which can maintain insurance against risk to avoid annual over-investment for stress-tolerator species. In addition, the slope aspect had a significant effect on the biomass allocation strategy between above- and belowground. Finally, both biotic and abiotic factors were responsible for the variation in total biomass. By integrating four species with different life forms, our study indicates that slope aspect strongly influences plant survival, growth, and propagation and thus regulates the ecological process in the alpine ecosystem. Therefore, more attention should be paid to the impact of slope aspect for grassland ecosystem conservation and management in the future.

## Data availability statement

The raw data supporting the conclusions of this article will be made available by the authors, without undue reservation.

## Author contributions

JW and YW raised scientific questions and designed the experiments. TZ, JG, and JW conducted the experiments. TZ analyzed the experimental data. TZ, JW, and LZ wrote the manuscript. All authors contributed to the article and approved the submitted version.
