# Supplementary material for: Divergent responses of plant functional traits and biomass allocation to slope aspects in four perennial herbs of the alpine meadow ecosystem
Source: Front Plant Sci. 2023 Mar 1;14:1092821. doi: 10.3389/fpls.2023.1092821 (PMC10016094; doi:10.3389/fpls.2023.1092821)
Supplement: Supplementary file 1 [file DataSheet_1.docx]

Table S1 The basic information for sampling

| Species | abbr | Mountain | Slope aspect | N |
| --- | --- | --- | --- | --- |
| *Bistorta macrophylla* | BISMAC | A | NE | 20 |
|  |  | A | SE | 20 |
|  |  | A | SW | 20 |
| *Bistorta vivipara* | BISVIV | A | NE | 30 |
|  |  | A | SE | 30 |
|  |  | A | SW | 15 |
| *Cremanthodium discoideum* | CREDIS | A | SE | 25 |
|  |  | A | SW | 20 |
| *Deschampsia littoralis* | DESLIT | A | SE | 15 |
|  |  | A | SW | 19 |
| *Bistorta macrophylla* | BISMAC | B | NE | 24 |
|  |  | B | SE | 23 |
|  |  | B | SW | 20 |
| *Bistorta vivipara* | BISVIV | B | NE | 20 |
|  |  | B | SE | 20 |
|  |  | B | SW | 20 |
| *Cremanthodium discoideum* | CREDIS | B | NE | 30 |
|  |  | B | SE | 20 |
|  |  | B | SW | 30 |
| *Deschampsia littoralis* | DESLIT | B | NE | 20 |
|  |  | B | SE | 30 |
|  |  | B | SW | 30 |

Note: abbr: abbreviation; NE: north-east; SE: south-east; SW: south-west; N: the number of samplings

Table S2 The PCA analysis for soil nutrients

| Abiotic factors | PC1 | PC2 | PC3 | PC4 | PC5 |
| --- | --- | --- | --- | --- | --- |
| Soil temperature (5 cm below ground, ℃) | 1.99 | 0.72 | 0.63 | -1.40 | 0.48 |
| Soil temperature (ground, ℃) | 0.36 | 0.46 | -2.43 | -0.92 | 0.04 |
| Bulk density (g cm^-3^) | 1.54 | 1.18 | 1.21 | -0.90 | -1.02 |
| Soil water content (%) | -2.29 | -0.99 | 0.07 | -0.72 | -0.58 |
| Nitrate nitrogen content (mg kg^-1^) | 1.86 | -0.49 | -1.62 | 0.69 | -0.52 |
| Ammonium nitrogen content (mg kg^-1^) | 0.03 | -2.29 | 0.84 | 1.06 | -0.03 |
| Soil organic carbon (g kg^-1^) | -0.36 | -2.34 | -0.28 | -1.15 | 0.27 |
| Total nitrogen (g kg^-1^) | 0.59 | -2.30 | 0.02 | -1.15 | -0.29 |
| Total phosphorus (g kg^-1^) | 2.42 | -0.80 | 0.59 | -0.07 | 0.47 |
| Available phosphorus (mg kg^-1^) | 2.28 | -0.98 | -0.28 | 0.86 | -0.27 |
| Eigenvalue | 3.78 | 2.96 | 1.64 | 1.29 | 0.33 |
| Proportion Explained | 0.38 | 0.30 | 0.16 | 0.13 | 0.03 |
| Cumulative Proportion | 0.38 | 0.67 | 0.84 | 0.97 | 1.00 |

Table S3 The intraspecific trait variation in four species

| Species | CV of ILA (%) | CV of ILM (%) | CV of SLA (%) | CV of Height (%) |
| --- | --- | --- | --- | --- |
| BISMAC | 49.89 | 88.05 | 28.12 | 18.87 |
| BISVIV | 42.38 | 45.40 | 19.86 | 18.05 |
| CREDIS | 38.40 | 43.88 | 24.80 | 24.10 |
| DESLIT | 44.98 | 56.59 | 26.86 | 24.36 |

Note: ILA, individual leaf area; ILM, individual leaf mass, SLA, specific leaf area, Height, plant height.

Table S4 Effect of slope aspect on functional traits of four species.

Note: Different letters indicate significant differences.

| Species | Mountain | Slope aspect | ILA  (cm^2^) | ILM  (g) | SLA  (cm^2^g^-1^) | Height  (cm) |
| --- | --- | --- | --- | --- | --- | --- |
| BISMAC | A | NE | 0.83±0.07b | 0.0022±0.0002b | 118.99±7.70 | 22.24±0.97 |
| BISMAC | A | SE | 1.05±0.11b | 0.0027±0.0003b | 103.84±11.31 | 21.74±0.65 |
| BISMAC | A | SW | 1.58±0.20a | 0.0056±0.0013a | 107.67±5.07 | 24.85±1.20 |
| BISVIV | A | NE | 5.33±0.306 | 0.0386±0.0028 | 140.61±2.81b | 25.92±0.84b |
| BISVIV | A | SE | 6.35±0.40 | 0.0435±0.0031 | 151.46±5.38ab | 29.36±0.71a |
| BISVIV | A | SW | 6.66±0.99 | 0.0419±0.0062 | 158.17±4.91a | 29.63±1.10a |
| CREDIS | A | SE | 6.38±0.54 | 0.0448±0.0040 | 146.47±5.53b | 14.55±0.51 |
| CREDIS | A | SW | 7.66±0.57 | 0.0471±0.0041 | 169.57±8.59a | 13.78±0.65 |
| DESLIT | A | SE | 1.35±0.15 | 0.0074±0.0011 | 194.24±9.33a | 45.07±1.95 |
| DESLIT | A | SW | 1.12±0.10 | 0.0068±0.0006 | 168.06±6.77b | 50.92±3.03 |
| BISMAC | B | NE | 1.40±0.12 | 0.0028±0.0003 | 119.95±5.32 | 24.70±0.97 |
| BISMAC | B | SE | 1.10±0.08 | 0.0030±0.0003 | 114.80±5.03 | 26.37±1.03 |
| BISMAC | B | SW | 1.08±0.11 | 0.0032±0.0004 | 104.77±5.18 | 23.66±0.59 |
| BISVIV | B | NE | 7.00±0.60 | 0.0446±0.0041 | 161.30±5.44b | 32.99±1.45 |
| BISVIV | B | SE | 7.82±0.66 | 0.0398±0.0040 | 203.02±6.84a | 32.08±1.24 |
| BISVIV | B | SW | 8.22±0.63 | 0.0430±0.0037 | 195.51±6.62a | 29.93±1.26 |
| CREDIS | B | NE | 7.87±0.48 | 0.0528±0.0034 | 151.26±3.36b | 14.74±0.60b |
| CREDIS | B | SE | 8.07±0.54 | 0.0529±0.0048 | 166.55±10.02b | 15.87±0.76ab |
| CREDIS | B | SW | 8.77±0.66 | 0.0478±0.0045 | 185.97±8.56a | 17.44±0.66a |
| DESLIT | B | NE | 1.24±0.09b | 0.0061±0.0005 | 212.97±11.39a | 49.38±2.75 |
| DESLIT | B | SE | 1.27±0.10b | 0.0064±0.0006 | 203.96±5.65a | 56.54±2.20 |
| DESLIT | B | SW | 1.56±0.12a | 0.0101±0.0009 | 165.20±7.86b | 50.22±1.99 |

Table S5 Effect of slope aspect on mass fraction of four species.

Note: Different letters indicate significant differences.

| Species | Mountain | Slope aspect | RMF  (%) | SMF  (%) | LMF  (%) | FMF  (%) |
| --- | --- | --- | --- | --- | --- | --- |
| BISMAC | A | NE | 60.10±2.29ab | 20.20±1.41 | 8.08±0.62a | 11.62±0.92 |
| BISMAC | A | SE | 67.07±2.62a | 18.18±2.64 | 4.65±0.55b | 10.10±1.06 |
| BISMAC | A | SW | 54.74±2.62b | 21.15±1.54 | 8.95±1.58a | 15.16±2.40 |
| BISVIV | A | NE | 74.08±1.26b | 13.41±1.18b | 11.03±0.68a | 1.48±0.11b |
| BISVIV | A | SE | 79.00±1.32a | 10.16±0.75c | 9.02±0.76b | 1.81±0.13b |
| BISVIV | A | SW | 60.90±2.38c | 24.49±1.84a | 12.26±0.79a | 2.35±0.19a |
| CREDIS | A | SE | 25.30±1.66 | 13.18±1.39 | 40.26±1.87 | 21.27±1.13b |
| CREDIS | A | SW | 23.05±1.48 | 13.03±0.95 | 38.81±1.41 | 25.11±1.51a |
| DESLIT | A | SE | 17.67±1.61a | 21.51±2.17 | 58.42±2.23 | 2.40±0.38b |
| DESLIT | A | SW | 12.62±1.42b | 25.77±2.82 | 56.10±3.02 | 5.51±0.94a |
| BISMAC | B | NE | 58.92±2.37 | 21.52±1.46ab | 5.52±0.46 | 14.03±0.96 |
| BISMAC | B | SE | 57.58±1.82 | 22.52±1.07a | 6.38±0.57 | 13.52±0.84 |
| BISMAC | B | SW | 64.57±2.29 | 18.03±1.33b | 6.12±0.73 | 11.28±0.83 |
| BISVIV | B | NE | 72.26±1.72a | 16.11±1.42c | 9.55±0.85a | 2.08±0.30b |
| BISVIV | B | SE | 62.45±1.39b | 24.03±1.06a | 10.14±0.70a | 3.38±0.38a |
| BISVIV | B | SW | 68.92±1.99a | 20.08±1.55b | 6.85±0.87b | 4.15±0.62a |
| CREDIS | B | NE | 21.89±1.84a | 10.75±0.66b | 46.54±2.13 | 20.82±1.48b |
| CREDIS | B | SE | 19.45±1.25ab | 10.99±0.83b | 46.57±2.39 | 22.99±1.89b |
| CREDIS | B | SW | 16.65±1.07b | 14.55±0.49a | 41.54±1.32 | 27.27±0.89a |
| DESLIT | B | NE | 40.88±2.07a | 13.80±1.60b | 42.63±2.29b | 2.69±0.30b |
| DESLIT | B | SE | 28.70±1.92b | 19.27±1.60a | 48.10±1.52a | 3.93±0.39a |
| DESLIT | B | SW | 24.50±1.28b | 21.92±1.63a | 49.42±1.62a | 4.16±0.39a |

| Species | Mountain | Slope aspect | Slope | Intercept | *P* | Test if the slope equals 1 (*p*) | Test for common slope (*p*) |
| --- | --- | --- | --- | --- | --- | --- | --- |
| BISMAC | A | NE | 1.07 | -0.12 | 0.064 | 0.064 | 0.411 |
| BISMAC | A | SE | 1.52 | -0.01 | **0.023** | **0.049** |  |
| BISMAC | A | SW | 1.10 | -0.02 | **0.001** | 0.592 |  |
| BISVIV | A | NE | 1.23 | -0.51 | **0.032** | 0.241 | 0.075 |
| BISVIV | A | SE | 0.89 | -0.57 | 0.103 | 0.538 |  |
| BISVIV | A | SW | 0.63 | -0.20 | **0.025** | 0.056 |  |
| CREDIS | A | SE | 0.60 | 0.32 | **0.036** | **0.010** | 0.486 |
| CREDIS | A | SW | 0.73 | 0.42 | **0.017** | 0.131 |  |
| DESLIT | A | SE | 1.05 | 0.73 | **0.032** | 0.844 | 0.105 |
| DESLIT | A | SW | 0.63 | 0.49 | **0.017** | **0**.**029** |  |
| BISMAC | B | NE | 1.31 | 0.05 | 0.087 | 0.178 | 0.388 |
| BISMAC | B | SE | 0.91 | -0.20 | **0.006** | 0.621 |  |
| BISMAC | B | SW | 1.17 | -0.16 | **0.014** | 0.430 |  |
| BISVIV | B | NE | 0.72 | -0.40 | **<0.001** | 0.058 | 0.120 |
| BISVIV | B | SE | 0.92 | -0.23 | **<0.001** | 0.629 |  |
| BISVIV | B | SW | 1.26 | -0.33 | **0.030** | 0.268 |  |
| CREDIS | B | NE | 0.66 | 0.44 | **0.031** | **0.019** | 0.108 |
| CREDIS | B | SE | 0.88 | 0.58 | **<0.001** | 0.429 |  |
| CREDIS | B | SW | 1.12 | 0.79 | **0.037** | 0.535 |  |
| DESLIT | B | NE | 0.90 | 0.15 | **<0.001** | 0.497 | 0.168 |
| DESLIT | B | SE | 0.63 | 0.24 | 0.098 | **0.015** |  |
| DESLIT | B | SW | 0.63 | 0.24 | **<0.001** | **0.001** |  |

Table S6 Effect of slope aspect on biomass allocation between above- and below-ground.

Table S7 The standardized estimates of structural equation models testing the impact of biotic and abiotic factors on total biomass for BISMAC.

| Response | Predictor | Crit.Value | Standard estimate | *P*-value |
| --- | --- | --- | --- | --- |
| Total biomass | Slope aspect | 0.951 | 0.140 | 0.344 |
| Total biomass | Height | 2.894 | 0.216 | 0.005 |
| Total biomass | ILM | 2.102 | 0.171 | 0.038 |
| Total biomass | ILA | 5.116 | 0.445 | 0.000 |
| Total biomass | SLA | -1.430 | -0.098 | 0.155 |
| Total biomass | PC1 | -0.232 | -0.030 | 0.817 |
| Total biomass | PC2 | -1.843 | -0.166 | 0.068 |
| Total biomass | PC3 | -0.682 | -0.050 | 0.496 |
| Height | Slope aspect | 1.935 | 0.345 | 0.055 |
| Height | PC1 | -0.994 | -0.160 | 0.322 |
| Height | PC2 | 2.775 | 0.306 | 0.006 |
| Height | PC3 | -3.148 | -0.291 | 0.002 |
| ILA | Slope aspect | 3.849 | 0.677 | 0.000 |
| ILA | PC1 | -2.859 | -0.454 | 0.005 |
| ILA | PC2 | 4.135 | 0.450 | 0.000 |
| ILA | PC3 | -1.550 | -0.141 | 0.124 |
| ILM | Slope aspect | 3.848 | 0.676 | 0.000 |
| ILM | PC1 | -2.303 | -0.365 | 0.023 |
| ILM | PC2 | 2.851 | 0.310 | 0.005 |
| ILM | PC3 | -0.026 | -0.002 | 0.979 |
| SLA | Slope aspect | -0.677 | -0.126 | 0.500 |
| SLA | PC1 | 0.011 | 0.002 | 0.992 |
| SLA | PC2 | 0.965 | 0.111 | 0.337 |
| SLA | PC3 | -0.308 | -0.030 | 0.759 |
| PC1 | Slope aspect | 13.327 | 0.766 | 0.000 |
| PC2 | Slope aspect | -5.033 | -0.411 | 0.000 |
| PC3 | Slope aspect | 1.399 | 0.124 | 0.164 |
| Partial bivariate correlations | | | | |
| ILA | ILM | 6.266 | 0.490 | 0.000 |
| PC2 | PC1 | 6.008 | 0.475 | 0.000 |
| ILA | Height | 4.427 | 0.370 | 0.000 |
| ILM | Height | 3.145 | 0.272 | 0.001 |

Table S8 The standardized estimates of structural equation models testing the impact of biotic and abiotic factors on total biomass for BISVIV.

| Response | Predictor | Crit.Value | Standard estimate | *P*-value |
| --- | --- | --- | --- | --- |
| total biomass | Slope aspect | 0.241 | 0.035 | 0.810 |
| total biomass | Height | 3.368 | 0.251 | 0.001 |
| total biomass | ILM | 1.114 | 0.298 | 0.267 |
| total biomass | ILA | -0.587 | -0.159 | 0.558 |
| total biomass | SLA | -1.101 | -0.140 | 0.273 |
| total biomass | PC1 | -3.340 | -0.472 | 0.001 |
| total biomass | PC2 | -1.488 | -0.143 | 0.139 |
| total biomass | PC3 | 1.809 | 0.149 | 0.073 |
| Height | Slope aspect | 1.734 | 0.304 | 0.085 |
| Height | PC1 | -1.219 | -0.199 | 0.225 |
| Height | PC2 | 1.983 | 0.229 | 0.050 |
| Height | PC3 | -4.778 | -0.402 | 0.000 |
| ILA | Slope aspect | 0.425 | 0.076 | 0.672 |
| ILA | PC1 | 1.118 | 0.186 | 0.266 |
| ILA | PC2 | 0.148 | 0.018 | 0.883 |
| ILA | PC3 | -2.596 | -0.223 | 0.011 |
| ILM | Slope aspect | 0.314 | 0.060 | 0.754 |
| ILM | PC1 | -0.185 | -0.033 | 0.854 |
| ILM | PC2 | 0.042 | 0.005 | 0.967 |
| ILM | PC3 | -0.428 | -0.039 | 0.669 |
| SLA | Slope aspect | 0.507 | 0.080 | 0.613 |
| SLA | PC1 | 1.742 | 0.256 | 0.084 |
| SLA | PC2 | 0.349 | 0.036 | 0.728 |
| SLA | PC3 | -5.592 | -0.424 | 0.000 |
| PC1 | Slope aspect | 13.254 | 0.754 | 0.000 |
| PC2 | Slope aspect | -5.169 | -0.409 | 0.000 |
| PC3 | Slope aspect | -0.735 | -0.064 | 0.464 |
| Partial bivariate correlations | | | | |
| ILA | ILM | 26.851 | 0.919 | 0.000 |
| PC2 | PC1 | 9.126 | 0.622 | 0.000 |
| ILA | Height | 2.703 | 0.229 | 0.004 |
| ILM | SLA | -3.953 | -0.325 | 0.000 |
| SLA | Height | 0.823 | 0.072 | 0.206 |

| Response | Predictor | Crit.Value | standard estimate | *P*-value |
| --- | --- | --- | --- | --- |
| Total biomass | Slope aspect | 0.597 | 0.137 | 0.552 |
| Total biomass | Height | 1.906 | 0.180 | 0.059 |
| Total biomass | ILM | -1.485 | -0.524 | 0.140 |
| Total biomass | ILA | 2.505 | 0.820 | 0.014 |
| Total biomass | SLA | -1.258 | -0.208 | 0.211 |
| Total biomass | PC1 | -0.592 | -0.107 | 0.555 |
| Total biomass | PC2 | 1.237 | 0.146 | 0.219 |
| Total biomass | PC3 | -1.326 | -0.163 | 0.187 |
| Height | Slope aspect | -1.311 | -0.315 | 0.193 |
| Height | PC1 | 3.050 | 0.559 | 0.003 |
| Height | PC2 | -1.746 | -0.212 | 0.083 |
| Height | PC3 | -0.530 | -0.069 | 0.597 |
| ILA | Slope aspect | 0.135 | 0.034 | 0.893 |
| ILA | PC1 | 0.925 | 0.178 | 0.357 |
| ILA | PC2 | 1.068 | 0.136 | 0.288 |
| ILA | PC3 | -0.680 | -0.092 | 0.498 |
| ILM | Slope aspect | 0.129 | 0.033 | 0.898 |
| ILM | PC1 | -0.095 | -0.019 | 0.925 |
| ILM | PC2 | 0.887 | 0.115 | 0.377 |
| ILM | PC3 | -0.830 | -0.115 | 0.409 |
| SLA | Slope aspect | 0.176 | 0.043 | 0.861 |
| SLA | PC1 | 1.751 | 0.323 | 0.083 |
| SLA | PC2 | 0.216 | 0.026 | 0.829 |
| SLA | PC3 | 0.291 | 0.038 | 0.772 |
| PC1 | Slope aspect | 12.381 | 0.745 | 0.000 |
| PC2 | Slope aspect | -4.894 | -0.404 | 0.000 |
| PC3 | Slope aspect | 7.843 | 0.577 | 0.000 |
| Partial bivariate correlations | | | | |
| ILA | ILM | 22.037 | 0.894 | 0.000 |
| PC2 | PC1 | 6.697 | 0.519 | 0.000 |
| PC3 | PC1 | -5.328 | -0.435 | 0.000 |
| ILA | Height | 3.499 | 0.302 | 0.000 |
| ILM | SLA | -5.616 | -0.453 | 0.000 |
| SLA | Height | 2.171 | 0.193 | 0.016 |

Table S9 The standardized estimates of structural equation models testing the impact of biotic and abiotic factors on total biomass for CREDIS.

| Response | Predictor | Crit.Value | Standard estimate | *P*-value |
| --- | --- | --- | --- | --- |
| Total biomass | Slope aspect | -2.595 | -0.550 | 0.011 |
| Total biomass | Height | 2.458 | 0.202 | 0.016 |
| Total biomass | ILM | 1.309 | 0.470 | 0.193 |
| Total biomass | ILA | -1.208 | -0.371 | 0.230 |
| Total biomass | SLA | 0.345 | 0.057 | 0.731 |
| Total biomass | PC1 | 0.448 | 0.073 | 0.655 |
| Total biomass | PC2 | 0.981 | 0.104 | 0.329 |
| Total biomass | PC3 | -0.750 | -0.092 | 0.455 |
| Height | Slope aspect | 2.190 | 0.515 | 0.031 |
| Height | PC1 | -1.278 | -0.228 | 0.204 |
| Height | PC2 | 2.030 | 0.241 | 0.045 |
| Height | PC3 | -3.212 | -0.433 | 0.002 |
| ILA | Slope aspect | -1.678 | -0.399 | 0.096 |
| ILA | PC1 | 2.513 | 0.454 | 0.013 |
| ILA | PC2 | -2.034 | -0.244 | 0.044 |
| ILA | PC3 | 0.806 | 0.110 | 0.422 |
| ILM | Slope aspect | -1.608 | -0.364 | 0.111 |
| ILM | PC1 | 3.241 | 0.557 | 0.002 |
| ILM | PC2 | -2.232 | -0.255 | 0.028 |
| ILM | PC3 | 1.656 | 0.215 | 0.101 |
| SLA | Slope aspect | -0.824 | -0.181 | 0.412 |
| SLA | PC1 | -1.037 | -0.173 | 0.302 |
| SLA | PC2 | 0.307 | 0.034 | 0.759 |
| SLA | PC3 | -1.529 | -0.193 | 0.129 |
| PC1 | Slope aspect | 12.441 | 0.762 | 0.000 |
| PC2 | Slope aspect | -4.316 | -0.378 | 0.000 |
| PC3 | Slope aspect | 7.381 | 0.572 | 0.000 |
| Partial bivariate correlations | | | | |
| ILA | ILM | 21.006 | 0.894 | 0.000 |
| PC2 | PC1 | 4.221 | 0.372 | 0.000 |
| PC3 | PC1 | -4.462 | -0.390 | 0.000 |
| ILM | SLA | -5.240 | -0.445 | 0.000 |

Table S10 The standardized estimates of structural equation models testing the impact of biotic and abiotic factors on total biomass for DESLIT.


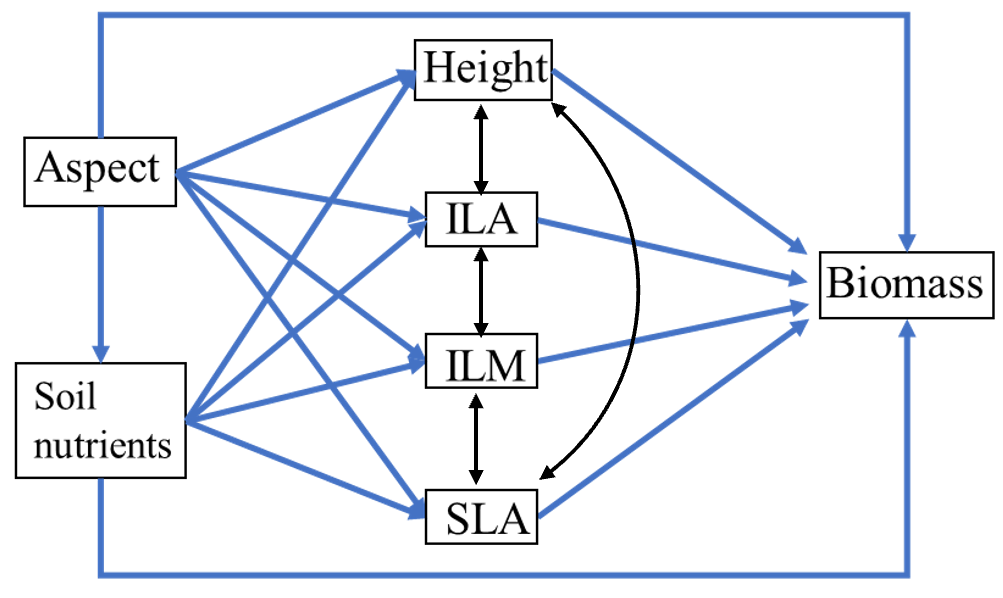


Figure S1 The conceptual model linking total biomass with the effects of abiotic and biotic factors. The arrows indicate the causal relationship. The structural equation model included soil nutrients (the first three axis of PCA for soil nutrients), slope aspect (Aspect), plant height (Height), individual leaf area (ILA), individual leaf mass (ILM), specific leaf area (SLA) and total biomass (Biomass). Black bi-directional arrows refer to significant correlations between variables.
